# Cell and Signal Components of the Microenvironment of Bone Metastasis Are Affected by Hypoxia

**DOI:** 10.3390/ijms17050706

**Published:** 2016-05-11

**Authors:** Paola Bendinelli, Paola Maroni, Emanuela Matteucci, Maria Alfonsina Desiderio

**Affiliations:** 1 Dipartimento di Scienze Biomediche per la Salute, Molecular Pathology Laboratory, Università degli Studi di Milano, 20133 Milano, Italy; paola.bendinelli@unimi.it (P.B.); emanuela.matteucci@unimi.it (E.M.); 2 Istituto Ortopedico Galeazzi, Scientific Institute for Research, Hospitalization and Health Care (IRCCS), 20161 Milano, Italy; paola.maroni@grupposandonato.it

**Keywords:** bone metastasis, hypoxic microenvironment, megakaryocytes, secreted protein acidic and rich in cysteine (SPARC), HIF-1, hepatocyte growth factor

## Abstract

Bone metastatic cells release bone microenvironment proteins, such as the matricellular protein SPARC (secreted protein acidic and rich in cysteine), and share a cell signaling typical of the bone metabolism controlled by Runx2. The megakaryocytes in the bone marrow engrafted by the metastases seem to be one of the principal microenvironment sources of the biological stimuli, implicated in the formation of an osteoblastic niche, and affecting metastasis phenotype and colonization. Educated platelets in the circulation might derive from megakaryocytes in bone metastasis. The evaluation of predictive markers in the circulating platelets might be useful for the stratification of patients for therapeutic purposes. The hypoxic environment in bone metastasis is one of the key regulators of the network of the biological soluble and structural components of the matrix. In bone metastatic cells under hypoxia, similar patterns of Runx2 and SPARC are observed, both showing downregulation. Conversely, hypoxia induces Endothelin 1, which upregulates SPARC, and these biological stimuli may be considered prognostic markers of bone metastasis in breast carcinoma patients.

## 1. Introduction

### 1.1. The Bone Marrow Is the “Metastatic Microenvironment”

Bone is a unique microenvironment made of calcified hydroxyapatite crystals forming a dense protein matrix, and it is interconnected with the bone marrow even more than their proximity implies. The bone marrow, in which osteoblast and osteoclast progenitors reside as well as hematopoietic stem cells (HSCs) in “niches”, is a common site of metastatic disease for breast and prostate carcinomas and multiple myeloma [1,2].

The hypoxic bone marrow (with 1%–7% O_2_) seems to enhance the growth of metastases in the skeleton [3,4]. Body tissues under pathophysiological conditions may adapt to limits well under the normoxia (20% O_2_) [5]. Tumor growth occurs also under 1% O_2_, and hypoxia regulates the metabolism including nutrient uptake, utilization, and waste removal [6].

The metastatic cells with bone tropism do not condition the bone marrow as extensively as the pulmonary metastasis does with the stroma, and the same mechanisms that govern the homing of HSCs in healthy individuals are co-opted by tumor cells [7]. The bone marrow is hospitable and conductive for bone metastasis formation, since circulating tumor cells (CTCs) establish a relationship with endosteal (osteoblastic) and vascular niches for HSCs [2].

### 1.2. Interaction of Megakaryocytes with Functional Niches in Bone Metastasis

Bone marrow is a source of mesenchymal stem cells that can give rise to cells of mesodermal lineages, such as osteocytes [8]. These mesenchymal stem cells play an important pro-tumorigenic role in the microenvironment of bone metastasis [9], and are known as bone marrow stromal fibroblasts expressing secreted protein acidic and rich in cysteine (SPARC)/osteonectin [10]. These stromal fibroblasts seem also to home to sites of tumorigenesis, and to integrate in primary tumor stroma [8]. SPARC belongs to the matricellular group of proteins, which are transiently secreted to the Extracellular matrix (ECM), without becoming part of the ECM mesh; mediating the adhesion between carcinoma cell surface and ECM, SPARC influences the epithelial-mesenchymal transition (EMT) [11].

Under physiological conditions, the “vascular niche” supports the expansion of hematopoietic lineages, and it appears especially critical for megakaryocyte function and platelet production. Evidence for a role of megakaryocytes in bone formation comes from data indicating that megakaryocytes express or secrete the bone matrix proteins osteocalcin, ostonectin, bone syaloprotein, and osteopontin [12]. Moreover, the megakaryocytes play important roles in the recovery of an “osteoblastic niche” following irradiation and bone marrow transplantation [2]. Notwithstanding the megakaryocyte functions, few studies deal with bone marrow histology and megakaryocyte implication in the recruitment of malignant cells, through the production of microenvironment stimuli, also under the influence of hypoxic conditions. In particular, the production of SPARC by megakaryocytes in bone metastasis has never been examined. Given the location of mature megakaryocytes at vascular sinusoids, they may be the first cells to physically encounter CTCs as they enter the bone marrow. Thus, a role of megakaryocytes in osteoblastic niche formation is strongly plausible, making the bone marrow hospitable for metastatic cells.

This article aims to provide a brief overview of hypoxia involvement in bone metastasis, influencing supportive cells, and biological stimuli production.

## 2. Hypoxia Affects the Response to Environmental Biological Stimuli Regulating Endothelin 1 (ET-1)/ET_A_R and Hepatocyte Growth Factor (HGF)/Met Receptor Axes in Bone Metastasis

The biological stimuli of paracrine and autocrine origin constitute a complex network in the bone metastasis microenvironment, important for dictating the various phases of colonization. Additionally, there is evidence of the interaction between biological and physical (hypoxia) stimuli in the transdifferentiation programs EMT and the reverse MET (epithelial phenotype) [13,14,15,16].

The hypoxic tumor microenvironment influences both the early and late stages of metastasis [17]. Hypoxia inducible factor-1 (HIF-1), an α-β heterodimer, is the master regulator of oxygen homeostasis that binds to Hypoxia responsive element (HRE) on target genes [4]. HIF-1α is stabilized by hypoxia as well as by oncogenes, despite oxygen, controlling HIF-1 activity that orchestrates the metabolism of neoplastic cells [4]. HIF-1 regulates EMT directly or indirectly. For example, HIF-1 plays a direct role on EMT transcription factors ZEB, Snail, and Twist. AXL receptor tyrosine kinase, a target gene of HIF-1, intervenes in EMT, invasion, and metastasis [17].

**Figure 1:**
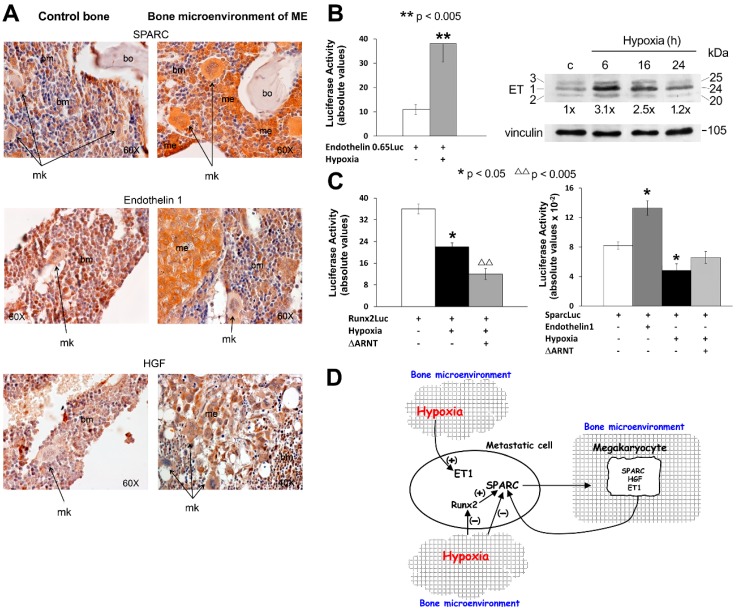
Production of biological stimuli by megakaryocytes in bone metastases, and their reciprocal regulation. (**A**) Immunohistochemistry of biological stimuli; (**B**) Effects of hypoxia on ET-1 transactivation and protein levels of the three isoforms of ET; (**C**) Runx2 and SPARC activities under hypoxia; (**D**) Cross-talk of metastasis and microenvironment through biological stimuli and involvement of hypoxic conditions.

Figure 1 shows original data on the production of biological stimuli under hypoxia. To clarify the function of megakaryocytes in the release of biological stimuli of the microenvironment, we assayed by immunohistochemistry the expression of SPARC, ET-1, and HGF in a xenograft model of bone metastasis (ME) (Figure 1A), prepared with human 1833 clone derived from invasive MDA-MB231 breast carcinoma cells [18]. The normal bone of a mouse was taken as control. Some of the biological stimuli in bone metastatic tissue were supposed to be responsive to hypoxia because of the presence of putative HRE-binding sites in the gene promoters. ET-1 and HGF promoters show 3 and 1 HREs (CGTG), respectively. In addition, ET-1 presents one different HIF-1 consensus sequence (CGTC) near the TATA box [19]. ET-1 is a critical player in tumor microenvironment, exerting multiple functions on migration and chemotaxis of neoplastic cells important for invasiveness and dissemination [20].

As shown in Figure 1A, the SPARC matricellular glycoprotein was remarkably expressed by the megakaryocytes (mk) of the bone marrow (bm), engrafted by the metastasis (me), much more than the megakaryocytes of normal bone marrow. A semiquantitative evaluation was done, and SPARC resulted (++++) in mk and me of the xenograft mice (*n* = 3). However, ET-1 seemed to be similarly expressed by megakaryocytes in normal and metastatic bone marrow (++). Notably, the bone metastasis stained very strongly for SPARC and ET-1 (++++), while the included bone (bo) was negative. As regards metastatic HGF, the immunohistochemical signal was observed prevalently in megakaryocytes and in the bone metastasis (++). In the normal bone marrow, the HGF signal was scarce (+) within cellular components. We cannot exclude that beyond megakaryocytes other mesenchymal and stem cells, supportive for metastases, produced the biological stimuli under observation. 

The megakaryocyte-derived factors including SPARC might influence the metastasis phenotype and colonization, being secreted to the ECM and forming the osteoblastic niche [21,22]. Bone micrometastases of breast cancer predominantly reside in a niche that exhibits features of osteogenesis [23], which might be responsible for the survival of micrometastases during quiescence and for their outgrowth [24].

We hypothesize that the packaging of SPARC, ET-1 and HGF into nascent platelets would modulate the “premalignant” platelet phenotype with systemic effects on CTCs, also favoring metastasis outgrowth [13]. Platelets play numerous functions in metastasis, and deepening the knowledge of the molecular mechanisms for platelet–CTC interactions is important to individuate patients with a high risk of metastasis [25]. Platelets seem to influence aggressive and mesenchymal phenotypes of CTCs by direct interaction. Breast carcinoma subtypes differ for molecular and clinical characteristics: The cells of luminal subtype B, usually associated with bone metastasis, when co-cultured with platelets activated by cathepsin K, show the upregulation of signaling pathways (Sonic Hedgehog) as well as of growth factors (TGF-β) and of bone-metastasis signature proteins (osteopontin) [26]. Cathepsin K, a cystein protease, is an attractive drug target being one of the cathepsins activated in a variety of cancers: It is localized in endosomes and lysosomes, and may be secreted in pericellular environments. An elevated platelet-lymphocyte ratio is associated with an increased risk of mortality in patients with ER+ or PR+ and Her2+ breast cancer [27]. An imbalance of the ratio of peripheral neutrophil/platelets to lymphocytes may provide an index of progression and prognosis to breast cancer patients, related to enhanced angiogenesis favoring metastasis. Different metastatic sites show various genetic and epigenetic alterations, and biopsies might not be representative. To overcome the problem of patient stratification for therapy, a potential role of tumor-educated blood platelets (TEPs) is reported [28]. The TEP-mRNA profile gives an actual idea of the status of metastatic lesions, useful for stratifying individual patients to appropriate molecular therapy.

We verified the role of ET-1 and hypoxia on the expression of metastatic SPARC. Hypoxia (administered as a gas mixture containing 5% CO_2_, 1% O_2_, and nitrogen balanced) was performed for 24 h [29]. As shown in Figure 1B, in hypoxic 1833 cells, ET-1 transactivation and protein level consistently increased: The ET-1 luciferase activity was evaluated by a transient transfection for 24 h of the construct containing the 650-bp promoter fragment [16]. Runx2 is a transcription factor involved in bone metabolism and tumor growth [30,31,32], and SPARC is a target gene of Runx2 [21], showing two HRE binding sites in the promoter. SPARC transactivation was downregulated under hypoxia, even if SPARC promoter does not present any HRE sequence. Thus, the effect of hypoxia on SPARCLuc seemed to be indirect, probably a consequence of Runx2 fall-down (Figure 1C). The dominant negative of the β subunit of HIF-1 (ΔARNT) further decreased Runx2 activity with respect to hypoxia alone, probably because HIF-1β is common to all members of the HIF-1 family [4]. Based on our data (from Figure 1C,D of the present paper and [19]), SPARC and Runx2 behaved similarly in response to different stimuli, and they were upregulated by ET-1 while being downregulated by hypoxia.

Notably, ET-1 and SPARC have diagnostic significance for patients, being highly expressed in dysplastic lesions adjacent to breast carcinoma and in pair-matched bone metastases [22]. The ET-1 receptor ET_A_R shows a pattern similar to that of the ligand, *i.e.*, ET_A_R expression is elevated in dysplasia and bone metastasis, with a characteristic distribution at the plasma membrane level while being low in primary breast carcinoma.

Finally, the data of our translational research suggest that ET-1 and HGF may create a signaling network in bone metastasis from breast carcinoma. In 1833 cells, HGF prevents the release of ET-1 and stabilizes the Met receptor; in parental MDA-MB231 cells, HGF enhances ET-1 release, and Met is rapidly downregulated [19,33]. Met is a HIF-1 target gene [34], with a possible anti-anoikis role [35]. In the 1833-xenograft model, the blockade of HGF with NK4, a competitive inhibitor that prevents HGF from binding to Met, prolongs mice survival; the inhibitory effect of NK4 on metastasis outgrowth is potentiated by the combination with a Src activity blockade with dasatinib. A critical molecular mechanism underlying the efficacy of the combined therapy is the triggering of autophagy failure [18]. The autophagy process seems protective for metastasis growth [36]. Additionally, HGF influences the function of WWdomain-containing oxidoreductase (Wwox), a regulator of Hippo pathway, in the control of the Twist program that is critical for mesenchymal-epithelial transition (MET) in bone metastatic cells [15].

## 3. Regulation of HIF-1 Activity in Metastatic Cells and in the Xenograft Model: The Influence on Pre-Metastatic Niche Formation

The role of HIF-1 transcription factor varies during breast carcinoma progression, and this might be also related to the complex regulation of HIF-1α subunit. Under hypoxia, HIF-1 is active in bone-metastatic 1833 cells opposite to invasive MDA-MB231 cells, even if HIF-1α is inducible in both cell lines. Since HIF-1β present in MDA-MB231 cells is downregulated by hypoxia, this might be one of the reasons why HIF-1 activity is hampered in invasive carcinoma cells [37]. It can be supposed that, in carcinoma cells, HIF-1α exerts functions independent of the regulation of HIF-1 activity.

We have identified some molecular events controlled by hypoxia, which are critical for bone metastasis. Hypoxia affects the expression of COX2 and of related genes like HIF-1α, through the release of HGF and TGF-β1. This signaling pathway seems to be triggered by the activation of autoregulatory loops, due to the binding to the respective Met and TGF-β1 receptors [37]. Another regulatory mechanism involves Wwox, which participates in HIF-1α nuclear translocation and stabilization, important for the expression of E-cadherin and the epithelial phenotype of hypoxic bone metastatic cells [38]. In fact, due to HIF-1α phosphorylation in response to moderate hypoxia (1% oxygen), corresponding to the oxygen tension in the bone marrow [3], the ternary complex HIF-1α/p53/HDM2 is not formed [39], but HDM2 interacts with Wwox, permitting HIF-1α stabilization, hyperphosphorylation, and translocation into the nuclei, indispensable for HIF-1 activity. In this condition, the E3-ubiquitin ligase function of HDM2 against HIF-1α is likely to be prevented. The *in vitro* data support those in specimens of human bone metastasis, showing HIF-1α almost exclusively at nuclear level, and the expression of COX2 and Wwox throughout the metastatic cell [37,38].

Hypoxia is involved both directly and indirectly in the induction of EMT, through Twist expression [17,35], favoring the dissemination of cells from the primary tumor site, while the revertant MET phenotype is important for bone colonization [15].

The expression of constitutively active or dominant negative form of HIF-1α in MDA-MB231 cells increases or decreases the metastasis and the blood vessel density in long bones; the HIF-1α knockdown by shRNA reduces the radiographic area of osteolytic lesions, decreases vessel density in bone metastasis, and increases survival time after the injection of breast cancer cells [40].

HIF-1 signaling is important for pre-metastatic niche formation via lysyl oxidase (LOX) (lung metastases) and tumor-lymphatic vessel cross-talk to support metastasis colonization in breast, melanoma, prostate, gastric, and colon cancer patients [17]. Analysis of the hypoxic secretome with MDA-MB231 and 1833 cells shows that LOX is one of the secreted proteins upregulated in 1833 cells and is associated with osteotropism and pre-metastatic niche formation [41,42]. These data support our results reported above. LOX is an HIF-1 target gene, and the tumor-secreted LOX in xenograft models is an important modulator of bone homeostasis, with increased osteoclast number, highlighting the potential of therapeutic strategies devised to hamper the formation of pre-metastatic lesions driven by osteoclasts and mediated by LOX in the bone [41].

Once the CTCs have homed at the metastatic niche, they must be capable of initiating a secondary growth. Cancer stem cells (CSC) are capable of asymmetric division, maintaining the exponential growth of the tumor bulk, and give metastases. Hypoxia influences the staminality of breast cancer cells together with the transcriptional co-activator with a PDZ-binding motif (TAZ), as demonstrated *in vivo* with a specific shRNA [4].

## 4. HIF-1 Activity in Human Bone Metastasis and Therapy

Immunohistochemical studies in tumor biopsies have linked increased HIF-1α protein levels with an increased risk of metastasis and mortality in lymph node-positive, lymph node-negative, Her2+, ER+, and unselected breast cancer patients [4]. Overexpression of HIF-1α was found to predict early relapse in breast cancer in a retrospective study with 745 patients [43].

Our immunohistochemical studies show that HIF-1α and TAZ are remarkably expressed in primary ductal breast carcinoma throughout the neoplastic cells, while in bone metastases their signals are elevated and localized in nuclei, consistent with the role of TAZ as a co-factor of HIF-1 [38]. In contrast, Wwox and E-cadherin are low in primary breast carcinoma and are re-expressed in bone metastasis [38], contributing to the MET phenotype.

In human bone metastases, Wwox, showing localization in the Golgi apparatus, might be implicated in HIF-1α translocation and stabilization leading to HIF-1 activity in bone metastasis [33]. It is known that in primary carcinomas HIF-1α stabilization depends on a hypoxic niche and/or oncogene activation; growth factors including HGF induce HIF-1α mRNA, transactivation, and protein expression through NF-κB and HIF-1 autoregulatory loop [4,14,44].

Chemotherapy against triple negative breast cancer (TNBC) is the object of various studies; these tumors are resistant to common anti-oestrogen and anti-Her2 therapies with the antibody trastuzumab. Chemotherapy induces HIF-1-dependent expression of IL-1 and IL-8 cytokines, which promote the phenotype of breast cancer stem cells and the multidrug resistance protein 1; these cells require increased anti-oxidant capacity to prevent oxidative stress and to maintain stemness important for tumor initiation. Targeting breast cancer stem cells by inhibiting HIF-1-regulated glutathione synthesis may improve outcome in TNBC [44].

Recent reviews summarize the new and important data on therapy of bone metastatic disease [45,46,47]. Drugs that inhibit osteoclast-mediated bone resorption (denosumab, bisphosphonates) are the standard of care for patients with skeletal metastasis from prostate, breast, and lung carcinomas. These types of tumors are highly vascularised and harbor hypoxic regions and disorganized vasculature: Sunitinib and sorafenib—small molecule inhibitors targeting the HIF-1-target gene VEGF—are currently in clinical trials for use in advanced breast cancer to hamper cell dissemination, but the access of these inhibitors to the bone marrow seems incomplete with the present administration routes [45].

Emerging therapies in bone metastasis target not only osteoclasts, but also osteoblasts and microenvironment [46]. In particular, these therapies are devised towards ET-1 and Wnt signaling pathways, important for osteosclerotic lesions and osteoblastogenesis typical of bone metastasis from prostate carcinoma [46]. We show in preclinical studies that these two signaling pathways are also important in osteolytic bone metastasis from breast carcinoma [19,33]: ET-1/ET_A_R and Wnt pathways are active in metastatic cells and in 1833-xenograft model conferring osteomimetic and staminal phenotypes to bone metastasis. Receptor antagonists of ET_A_R like atrasentan and zibotentan, and sclerostin—an osteocyte-derived inhibitor of osteoblast activity in phase II study—would contribute significantly to a reduction in the frequency of metastatic outgrowth in the skeletons of patients with different primary carcinomas, forming both osteoblastic and osteolytic metastases. A recent study with tasquinimod demonstrates a reduced establishment of bone metastasis from prostate cancer through a combination of effects on the pre-metastatic niche, homing, immunological status, and osteogenesis [48].

New promising therapies for bone metastasis from various primary tumors target the mTOR pathway [47]. mTOR is an important regulator of cell signaling in physiological processes, such as growth, survival, and autophagy, and in different pathologies interesting also the bone. In fact, mTOR signaling regulates osteoclastogenesis and osteoblast differentiation. The mTOR blockade with rapamycin inhibits metastasis-associated osteolytic disease from breast carcinoma and neuroblastoma. The analogue everolimus not only inhibits osteoclastogenesis but also cathepsin K, the main collagen-degrading enzyme in osteoclasts, suggesting an effect on bone microenvironment cells [47]; an involvement of reactive oxygen species cannot be excluded because stressful conditions affect mTORC1 [49,50]. However, in *in vivo* models, rapamycin inhibits metastasis growth and angiogenesis, probably increasing microenvironment hypoxia [51]. Everolimus is also promising in combination with aromatase inhibitor exemestane, reducing the incidence of new bone metastases and pre-existing metastasis progression in patients with breast carcinoma [52,53].

In conclusion, the knowledge of the biology of bone metastasis is fundamental in devising effective therapies, targeting microenvironmental stimuli that influence the phenotype, the organotropism, and the osteoblastic niche formation.

## Abbreviations

SPARC	Secreted protein acidic and rich in cysteine
HSCs	Hematopoietic stem cells
CTCs	Circulating tumor cells
EMT	Epithelial-mesenchymal transition
ET-1	Endothelin-1
HGF	Hepatocyte growth factor
HIF-1	Hypoxia inducible factor-1
Wwox	WWdomain-containing oxidoreductase
MET	Mesenchymal-epithelial transition
LOX	Lysyl oxidase
CSC	Cancer stem cells
TAZ	Transcriptional co-activator with PDZ-binding motif
TNBC	Triple negative breast cancer
TEPs	Tumor-educated blood platelets
